# Supplementary material for: Occurrence of late-apoptotic symptoms in porcine preimplantation embryos upon exposure of oocytes to perfluoroalkyl substances (PFASs) under in vitro meiotic maturation
Source: PLoS One. 2022 Dec 28;17(12):e0279551. doi: 10.1371/journal.pone.0279551 (PMC9797085; doi:10.1371/journal.pone.0279551)
Supplement: Attachment — Submitted filename: Reviewer Attachment for Manuscript No. PONE-D-22-27248 PLoS One 4th November 2022.docx [file pone.0279551.s002.docx]

4^th^ November, 2022

Review of the Manuscript No. **PONE-D-22-27248**, by A. Leclercq et al., entitled: “**Apopotosis in porcine blastocysts upon exposure to perfluoroalkyl substances (PFASs) during oocyte maturation *in vitro***” that is intended to be published as the **Research Article** in ***PLoS One***

**(separate Microsoft Word file as Reviewer Attachment for Manuscript No. PONE-D-22-27248 PLoS One 4th November 2022 that includes Comments to the Authors is also uploaded)**

Taking into consideration research highlight, contribution of the Authors to the progress in the research area, thorough manner of data presentation, relatively well writing in English, abundance of Materials and Methods, Results, Tables and Figures (diligent tabular and graphic/photographic documentation), the quality of this paper deserves praise and merits my support. The Authors have received the high scores from me for the originality, importance of the work and the scientific value of their paper. In my opinion, the current paper provides insightful interpretation of topical and coming trends in assisted reproductive technologies (ARTs) targeted at recognizing the ectopic multi-functional molecular factors (perfluorooctane sulfonic acid; PFOS and perfluorohexane sulfonic acid; PFHxS) that represent the group of perfluoroalkyl substances (PFASs) and act as endocrine disruptors, inducers of gameto- and embryotoxicity and promoters/agonists of apoptotic cell death. The use of these factors for ARTs can contribute to attenuation of cytological quality of porcine IVF-derived embryos by augmented incidence of TUNEL-positive (i.e., late-apoptotic) cells in the blastocysts generated under the *in vitro* culture conditions. For all those reasons, I recommend the Editorial Board to allow for publication of this interesting paper in *PLoS One*, after the minor revision of the manuscript will have been completed by the Authors and provided that the Authors are ready to consider all the Reviewer comments indicated below:

1) The current title of the manuscript:

**“Apopotosis in porcine blastocysts upon exposure to perfluoroalkyl substances (PFASs) during oocyte maturation *in vitro*”**

should be re-edited to one of the following versions:

a) “**Occurrence of late-apoptotic symptoms in porcine *ex vivo*-fertilized embryos upon exposure of oocytes to perfluoroalkyl substances (PFASs) under the conditions of *in vitro* meiotic maturation”**
b) “**Exposure of porcine oocytes to perfluoroalkyl substances (PFASs) during their *in vitro* meiotic maturation triggers late-apoptotic events in the ex vivo-fertilized embryos”**
2) In my opinion, there is a lack of finalizing paragraph (including future directions and goals) within the Conclusion section. Therefore, missing details at the end of Conclusion section and missing research article citations and related References are required to be added according to the Reviewer comments shown below:

Considering the aforementioned fact, the following sentences have to be added between the lines 366 and 367 of the Conclusion section (on the page 16) as indicated below:

Finally, the current research provides insightful interpretation of coming trends in assisted reproductive technologies (ARTs) targeted at recognizing the ectopic multi-functional molecular factors (PFOS and PFHxS) that represent a family of PFASs and act as endocrine disruptors, inducers of gameto- and embryotoxicity and promoters/agonists of apoptotic cell death. The use of these factors for ARTs can contribute to attenuation of cytological quality of porcine IVF-derived embryos by augmented incidence of TUNEL-positive (i.e., late-apoptotic) cells in the blastocysts generated under the *in vitro* culture conditions. The results of these investigations might be extrapolated to studies focused on the in vitro production (IVP) of porcine and other mammalian embryos that have been generated by such innovative assisted reproductive technologies (ARTs) as intracytoplasmic sperm injection (ICSI)-mediated IVF (46–49) and somatic cell nuclear transfer (SCNT)-mediated cloning (50–55).

3) The following 10 References have to be added and cited in the text of manuscript (according to the re-editions required by Reviewer in the above-listed comment 1):

46. Okada T, McIlfatrick S, Hin N, Aryamanesh N, Breen J, St John JC. Mitochondrial supplementation of Sus scrofa metaphase II oocytes alters DNA methylation and gene expression profiles of blastocysts. Epigenetics Chromatin. 2022;15(1):12. doi: 10.1186/s13072-022-00442-x

47. Gorczyca G, Wartalski K, Romek M, Samiec M, Duda M. 2022. The Molecular Quality and Mitochondrial Activity of Porcine Cumulus-Oocyte Complexes Are Affected by Their Exposure to Three Endocrine-Active Compounds under 3D In Vitro Maturation Conditions. Int J Mol Sci. 2022;23(9): 4572. doi: 10.3390/ijms23094572

48. Tsampras N., Kollmann M, Craciunas L. Recombinant versus bovine hyaluronidase for oocyte denudation before intracytoplasmic sperm injection: a systematic review and meta-analysis. J Obstet Gynaecol. 2022;42(2): 301–5. doi: 10.1080/01443615.2021.1893670

49. Hernández-Pichardo JE, Ducolomb Y, Romo S, Kjelland ME, Fierro R, Casillas F, Betancourt M. Pronuclear formation by ICSI using chemically activated ovine oocytes and zona pellucida bound sperm. J Anim Sci Biotechnol. 2016;7:65. doi: 10.1186/s40104-016-0124-6

50. Wiater J, Samiec M, Skrzyszowska M, Lipiński, D. Trichostatin A-Assisted Epigenomic Modulation Affects the Expression Profiles of Not Only Recombinant Human α1,2-Fucosyltransferase and α-Galactosidase A Enzymes But Also Galα1→3Gal Epitopes in Porcine Bi-Transgenic Adult Cutaneous Fibroblast Cells. Int J Mol Sci. 2021;22(3): 1386. doi: 10.3390/ijms22031386

51. Skrzyszowska M, Smorąg Z, Słomski R, Kątska-Ksiażkiewicz L, Kalak R, Michalak E, Wielgus K, Lehmann J, Lipiński D, Szalata M, Pławski A, Samiec M, Jura J, Gajda B, Ryńska B, Pieńkowski M. Generation of transgenic rabbits by the novel technique of chimeric somatic cell cloning. Biol Reprod. 2006;74(6): 1114–20. doi: 10.1095/biolreprod.104.039370

52. Assareh N, Shahemabadi M, Varnosfaderani SR, Jafarpour F, Hajian M, Nasr-Esfahani MH. Sequential IVM by CNP preincubation and cooperating of PGE2 with AREG enhances developmental competence of SCNT reconstructs in goat. Sci Rep. 2022;12(1): 4243. doi: 10.1038/s41598-022-08238-5

53. Samiec M, Skrzyszowska M. Extranuclear Inheritance of Mitochondrial Genome and Epigenetic Reprogrammability of Chromosomal Telomeres in Somatic Cell Cloning of Mammals. Int J Mol Sci. 2021;22(6): 3099. doi: 10.3390/ijms22063099

54. Petersen B, Kurtz S. Generation of Pigs that Produce Single Sex Progeny. Methods Mol Biol. 2022;2495: 275–93. doi: 10.1007/978-1-0716-2301-5_15

55. Opiela J, Samiec M, Romanek J. *In vitro* development and cytological quality of inter-species (porcine→bovine) cloned embryos are affected by trichostatin A-dependent epigenomic modulation of adult mesenchymal stem cells. Theriogenology. 2017;97: 27–33. doi: 10.1016/j.theriogenology.2017.04.022

4) Finally, the manuscript requires to be slightly revised/corrected for English-language grammar, spelling and style.

**General Comment of the Reviewer:**

**Before the manuscript will have been accepted for publication in *PLoS One*, it requires the minor revision (according to all the remarks and suggestions of the Reviewer) and confirmation for the correctness of changes that will have been made by the Authors in the re-edited and resubmitted version of their paper.**
